# A Machine Learning Model to Predict the Triple Negative Breast Cancer Immune Subtype

**DOI:** 10.3389/fimmu.2021.749459

**Published:** 2021-09-17

**Authors:** Zihao Chen, Maoli Wang, Rudy Leon De Wilde, Ruifa Feng, Mingqiang Su, Luz Angela Torres-de la Roche, Wenjie Shi

**Affiliations:** ^1^Department of Urology, University of Freiburg, Freiburg, Germany; ^2^Department of Breast Surgery, Obstetrics and Gynecology Hospital of Fudan University, Shanghai, China; ^3^University Hospital for Gynecology, Pius-Hospital, University Medicine Oldenburg, Oldenburg, Germany; ^4^Breast Center of The Second Affiliated Hospital of Guilin Medical University, Guilin, China; ^5^Department of Urology, Zigong Hospital, Affiliated to Southwest Medical University, Zigong, China

**Keywords:** TNBC (triple negative breast cancer), immune checkpoint blockade, immune subtype, web server, TCGA

## Abstract

**Background:**

Immune checkpoint blockade (ICB) has been approved for the treatment of triple-negative breast cancer (TNBC), since it significantly improved the progression-free survival (PFS). However, only about 10% of TNBC patients could achieve the complete response (CR) to ICB because of the low response rate and potential adverse reactions to ICB.

**Methods:**

Open datasets from The Cancer Genome Atlas (TCGA) and Gene Expression Omnibus (GEO) were downloaded to perform an unsupervised clustering analysis to identify the immune subtype according to the expression profiles. The prognosis, enriched pathways, and the ICB indicators were compared between immune subtypes. Afterward, samples from the Molecular Taxonomy of Breast Cancer International Consortium (METABRIC) dataset were used to validate the correlation of immune subtype with prognosis. Data from patients who received ICB were selected to validate the correlation of the immune subtype with ICB response. Machine learning models were used to build a visual web server to predict the immune subtype of TNBC patients requiring ICB.

**Results:**

A total of eight open datasets including 931 TNBC samples were used for the unsupervised clustering. Two novel immune subtypes (referred to as S1 and S2) were identified among TNBC patients. Compared with S2, S1 was associated with higher immune scores, higher levels of immune cells, and a better prognosis for immunotherapy. In the validation dataset, subtype 1 samples had a better prognosis than sub type 2 samples, no matter in overall survival (OS) (p = 0.00036) or relapse-free survival (RFS) (p = 0.0022). Bioinformatics analysis identified 11 hub genes (LCK, IL2RG, CD3G, STAT1, CD247, IL2RB, CD3D, IRF1, OAS2, IRF4, and IFNG) related to the immune subtype. A robust machine learning model based on random forest algorithm was established by 11 hub genes, and it performed reasonably well with area Under the Curve of the receiver operating characteristic (AUC) values = 0.76. An open and free web server based on the random forest model, named as triple-negative breast cancer immune subtype (TNBCIS), was developed and is available from https://immunotypes.shinyapps.io/TNBCIS/.

**Conclusion:**

TNBC open datasets allowed us to stratify samples into distinct immunotherapy response subgroups according to gene expression profiles. Based on two novel subtypes, candidates for ICB with a higher response rate and better prognosis could be selected by using the free visual online web server that we designed.

## Introduction

Breast cancer (BC) is the most prevalent cancer in women worldwide, and the number of new cases diagnosed was 2.3 million in 2020 (1). The 5-year relative survival rate for BC patients is about 90% in America (2). However, as an aggressive subgroup of breast cancer, triple-negative breast cancer (ER−, PR−, and Her2− negative, TNBC) comprises approximately 15%–20% of BC incidence (3). The 5-year survival rate of the metastatic TNBC patient is <30% (4). Because of the chemoresistance and poor prognosis (5), novel therapeutic drugs are necessary for TNBC patients.

Recently, immune checkpoint blockade (ICB), especially Pembrolizumab (PD-1 antibody) and Atezolizumab (PD-L1 antibody), have achieved great success in multiple cancer types such as melanoma (6), breast cancer (7), and bladder cancer (8). The Food and Drug Administration (FDA) granted the combination of chemotherapy and Pembrolizumab for the treatment of PD-L1^+^ metastatic TNBC patients (9). This approval was based on a randomized, double-blind, phase III, TNBC study (NCT02819518), in which the progression-free survival (PFS) was significantly higher in the Pembrolizumab–Chemotherapy arm (9.7 months) than in the placebo arm (5.6 months) (hazard ratio, 0.65; 95% confidence interval, 0.49–0.86, *p* = 0.0012) (10). Moreover, in another clinical trial (NCT02425891), PFS was 7.2 months in the Atezolizumab–Chemotherapy group, which was better than 5.5 months in the Placebo–Chemotherapy arm (hazard ratio, 0.80; 95% confidence interval, 0.69–0.92, *p* = 0.002) (11).

Although immunotherapies are promising drug candidates for patients with TNBC (12), there are some limitations including low response rates, the risk of side effects, and the lack of robust biomarkers. The approximate response rate to ICB was 20% in most solid tumors (13). Without candidate selection, only 10% of TNBC patients in the Atezolizumab–Chemotherapy group achieved a complete response (11). The rates of adverse reactions including hypothyroidism, colitis, and pneumonitis were increased with the PD-1 antibodies treatment group than that with the control group (14). PD-L1/PD-1 levels and tumor mutational burden (TMB) could potentially indicate the immunotherapy effectiveness. However, PD-L1 is a dynamic biomarker (15) and lacks a standard detection method (16). Moreover, the objective response rate (ORR) for PD-L1^+^ TNBC patients was only 17% (17). The detection of TMB is challenging, expensive, and time consuming (18). These limitations suggest that an effective, efficient, convenient method to select the candidate for immunotherapies is needed.

In the current study, we aim at providing a web server that could contribute to the clinical use of immunotherapies by small counts of genes. A total of 934 samples from eight TNBC cohorts were selected for the construction of immune subtypes. Two immune subtypes (named S1 and S2) were identified, and the subtype S1 was correlated with the better prognosis, biomarkers for ICB, and immune-related pathways. We, therefore, hypothesized that subtype S1 patients are more likely to respond to ICB. A total of 313 TNBC samples from the Molecular Taxonomy of Breast Cancer International Consortium (METABRIC) dataset (validation dataset) demonstrated that subtype S1 was correlated with a better prognosis. After validation by the data from four independent immunotherapy trials, S1 patients demonstrated a higher response rate and better prognosis. Eleven differentially expressed genes (DEGs) were selected for the immune subtype prediction model building. After that, a convenient and free web server was provided based on the constructed model. Overall, our study might contribute to explore the heterogeneity among TNBC patients and provide a way to identify the appropriate patient for the immunotherapies.

## Materials and Methods

### Data Obtaining and Preprocessing

Eight TNBC datasets were used as training sets for immune subtype identification. One independent dataset was used to verify the correlation between immune subtypes and prognosis. Four independent datasets containing patient immunotherapy information were used to assess the correlation between immune subtypes and ICB response. The training datasets included GSE18864 (38 TNBC samples) (19), GSE58812 (107 TNBC samples) (20), GSE76124 (198 TNBC samples) (21), GSE76250 (165 TNBC samples) (22), GSE83937 (131 TNBC samples) (23), GSE95700 (57 TNBC samples) (24), GSE106977 (119 TNBC samples) (25), and The Cancer Genome Atlas (TCGA) (116 TNBC samples) (26). The validation dataset1 was the METABRIC dataset. A total of 313 ER- and HER2-negative breast cancer from METABRIC were selected, since the overall survival (OS), relapse-free survival (RFS) information and expression matrix were available. The validation dataset2 included GSE78220 study containing 28 melanoma samples (PD-1 antibody) (27), GSE35640 study containing 65 lung cancer samples (MAGE-A3 immunotherapy) (28), IMvigor210 study containing 348 bladder cancer samples (PD-L1 antibody) (29), and GSE91061 study containing 51 melanoma samples treated with immune checkpoint blockade (30). The gene-expression matrix and the survival results of these training and validation datasets were obtained from TCGA, cBioPortal, and Gene Expression Omnibus (GEO). The Masked Somatic Mutation data (mutect2) of TNBC samples from the TCGA-TNBC dataset were obtained. The TMB value of each sample is equal to the total mutation frequency/the number of megabases of the human exome.

### Immune Cell Levels and Immune Scores

Single Sample Gene Set Enrichment Analysis (ssGSEA) is a tool to calculate the levels of immune cells by expression data of the immune-cells-specific genes. We collected the immune-cells-specific-genes data from a previous article (31). The levels of immune cells were estimated by the “GSVA” package in the R language (32). Estimation of Stromal and Immune cells in Malignant Tumor Tissues Using Expression Data (ESTIMATE) is a method to predict immune scores, stromal scores, and tumor purity in tumor tissues by gene expression data (33).

### Identification of TNBC Immune Subtypes

Consensus clustering (CC) is an unsupervised clustering tool to find the unidentified subgroups/subtypes by the gene expression data (34), and the “ConsensusClusterPlus” package was selected to do CC analysis (35). The CC parameters including “maxK,” “clusterAlg,” and “distance” were selected as “6,” “hc,” and “Pearson.” The optimized immune subtype number (K) was selected by tracking plot, cumulative density function, and relative change in area under cumulative density function (36).

### Differentially Expressed Genes Screening

Since the samples were divided into two immune subtypes, we performed the DEGs analysis to identify the genetic differences between the two immune subtypes. The DEGs analysis by “limma” package from R language was performed in each dataset (37). Then, we screened the DEGs for each dataset to obtain those with the *p* < 0.05 and |log2(fold change)| > 0.5.

### Robust Rank Aggregation Analysis

To integrate the screened DEGs from these 8 expression datasets, the robust rank aggregation (RRA) method that could reduce the bias among datasets was used. RRA method could estimate the ranking of DEGs lists. If a DEG ranked the highest in all lists, it will be regarded as a robust DEG with the smallest *p*-value. Multiple studies select RRA to integrate DEG results from different datasets, since it is robust to errors and noise (38). Significance scores for all genes were provided by RRA, and only the statistically relevant genes were retained (39). RRA was performed by the “RobustRankAggreg” package in R language to obtain the robust DEGs among different datasets (39). Genes with |log2(fold change)| > 0.5 and *p* < 0.05 were selected as robust DEGs.

### Gene Set Enrichment Analysis

Gene sets including Kyoto Encyclopedia of Genes and Genomes (KEGG), REACTOME, and Biological Processes were downloaded from the MSigDB database (40). The gene sets were selected based on the criteria: “GeneNumber > 15.” R package “fgsea” was used to perform GSEA and visualize the top enriched gene sets. The gene sets with *p* < 0.05 were considered statistically significant.

### Weighted Gene Coexpression Network Analysis

To identify the hub genes/proteins for tumor grades, the DEGs expression data from TCGA-TNBC were chosen to be analyzed by the “WGCNA” package in R language (41). The input data for Weighted Gene Coexpression Network Analysis (WGCNA) is the expression data of tumor samples and their correspondent clinical information. First, outliers were identified by cluster analysis and then removed. Second, screening the best value of soft threshold power β was crucial to guarantee a scale-free network. Then, the parameters such as minModulesize (42) and mergeCutHeight (0.25) were set to identify the potential modules. Moreover, the correlations between modules and clinical traits were calculated to select the modules. Lastly, the gene significance (GS) of the gene in the module was calculated, since it represents the correlation between gene and sample.

### Identification of Hub Genes

Protein–protein interaction (PPI) network of the selected module was constructed to select the hub genes/proteins. All the genes in the selected module were uploaded to the STRING website, and only the interactions that came from experiments were retained. Besides, the interactions were characterized as significant interactions when their confidence value was >0.7. After obtaining the PPI network, the genes/proteins were characterized as hub genes/proteins when their degree value was >10.

### Immune Subtypes Prediction Model

Random forest (RF) from the “caret” package in R language was trained to build the immune subtype prediction model (43). The expression data of hub genes from the TCGA-TNBC cohort was used in the process of model training. First, the gene expression data were randomly divided into two datasets, the training dataset (70%) and the testing dataset (30%). Second, grid search with fivefold cross-validation was employed for RF model training. Then, the prediction ability of the constructed RF model was evaluated by the testing dataset. Finally, four datasets (GSE78220, GSE35640, GSE91061, and IMvigor210) were selected as the independent validation dataset to verify the correlation of immune subtype and ICB response.

### Immune Subtype Prediction Web Server

Shiny application from the R “shiny” package is a tool to construct open and free web servers (44). There is no limit for this constructed web server to be used by any users. The constructed web server was tested in different computer systems including Linux, Windows, and macOS, and browsers such as Chrome, Firefox, and Internet Explorer.

## Results

### Calculation of Immune Cells Levels

**Figure 1:**
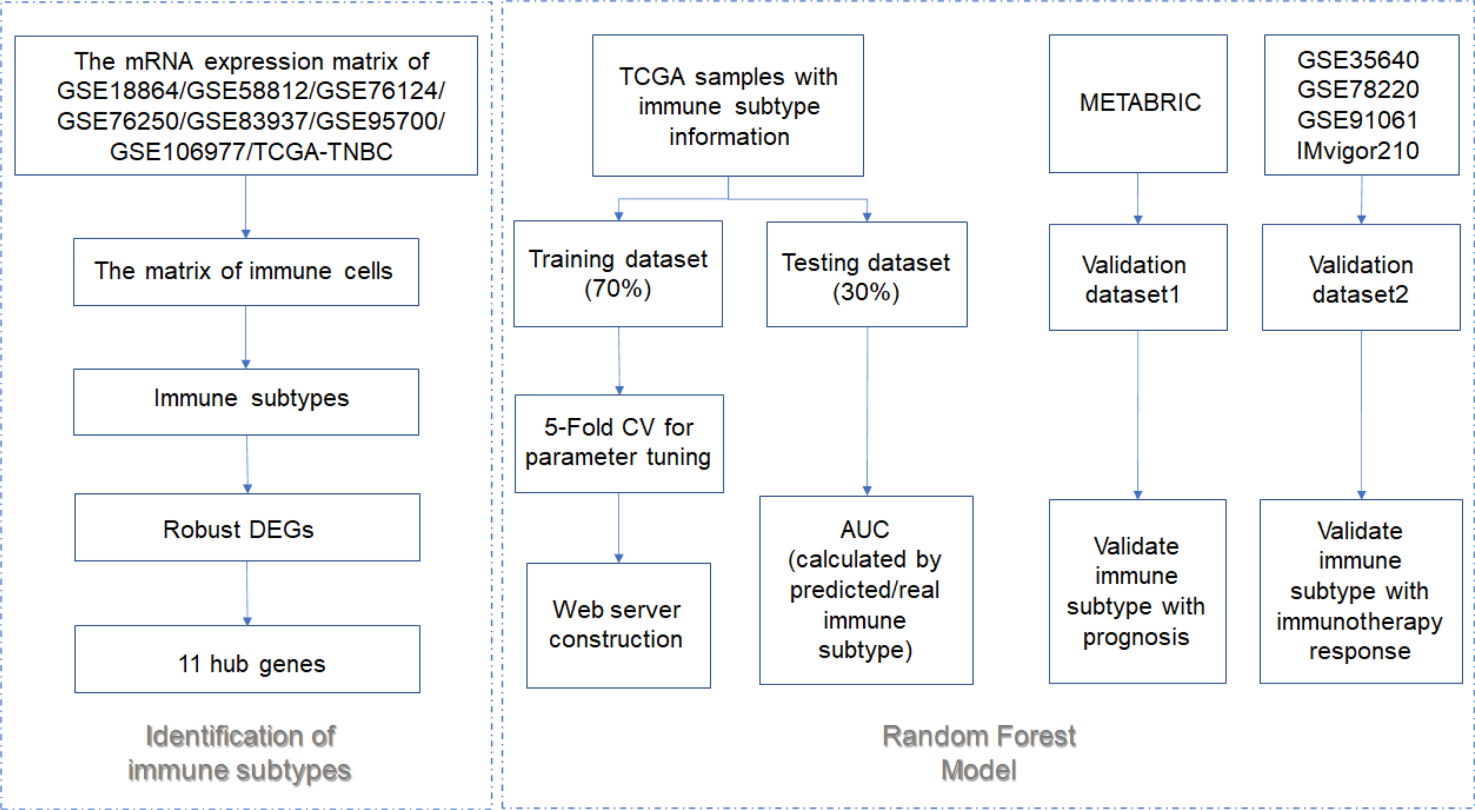
The flowchart of this study.

**Figure 2:**
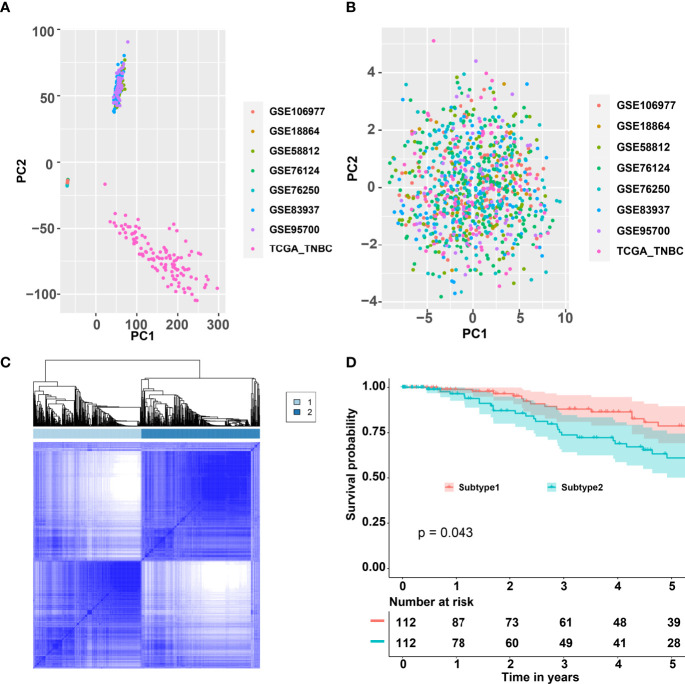
Consensus clustering for the TNBC by combining eight datasets GSE18864, GSE58812, GSE76124, GSE76250, GSE83937, GSE95700, GSE106977, and TCGA. **(A)** PCA of the expression matrix of eight different datasets. **(B)** PCA of the levels of the immune cells of eight different datasets. **(C)** Consensus matrix heatmap plots when k = 2. **(D)** Five-year Kaplan–Meier curves for OS of TNBC patients stratified by the immune subtypes. *p*-value was calculated by the log-rank test among subtypes. TNBC, triple-negative breast cancer; PCA, principal component analysis; TCGA, The Cancer Genome Atlas; OS, overall survival.

The workflow is plotted in **Figure 1**. Eight datasets, namely, GSE18864 (38 TNBC samples), GSE58812 (107 TNBC samples), GSE76124 (198 TNBC samples), GSE76250 (165 TNBC samples), GSE83937 (131 TNBC samples), GSE95700 (57 TNBC samples), GSE106977 (119 TNBC samples), and TCGA (116 TNBC samples) were obtained and used. The gene-expression profiles of these datasets were then transformed into the data of immune cell levels by ssGSEA. Before the transformation, principal component analysis (PCA) results demonstrated an obvious batch effect (**Figure 2A**). After the transformation, PCA results illustrated that the batch effect was successfully removed (**Figure 2B**).

### Immune Subtypes Among TNBC Patients

CC analysis was performed on the data of immune cell levels from 931 TNBC patients. Two immune subtypes named S1 and S2 were recognized (**Figure 2C**). The tracking plot indicated that “two” was the optimal immune subtype number (**Supplementary Figure 1A**). The area under the cumulative distribution function (CDF) curve and its relative change indicated that 4 was the best value for immune subtype number (**Supplementary Figures 1B, C**). Two immune subtypes, but not four immune subtypes, would contribute to the binary classification model building. As shown in **Figure 2D**, the OS rates of S1 samples were significantly better than that of the S2 samples. Moreover, the PFS of S1 samples were significantly better than that of the S2 samples (**Supplementary Figure 2A**).

**Figure 3:**
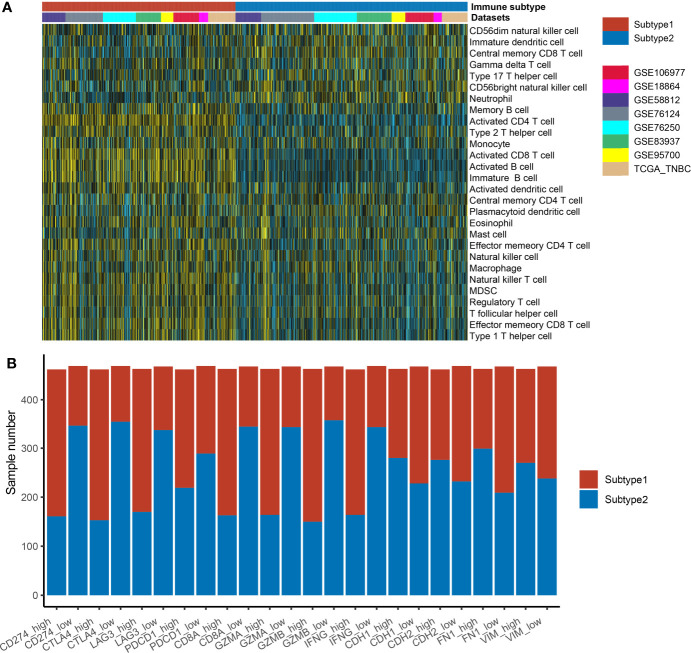
The distribution of immune cell enrichment scores and immune-related markers in two different immune subtypes. **(A)** The immune cell enrichment scores in two subtypes are displayed by heatmap. **(B)** The immune-related markers in two different immune subtypes are displayed by the boxplot. The expression values of these markers in each dataset were transformed into “high” or “low” by the median value of the marker. Then, the correlation of immune subtypes (subtype 1 or subtype 2 groups) and expression groups (high or low groups) was tested by the Fisher’s test.

S1 samples demonstrated the high levels of adaptive immune cells including activated B cells, activated CD8 T cells, and activated CD4 T cells, while S2 samples showed higher levels of neutrophils and type 17 helper cells (**Figure 3A**). The association of the identified immune subtypes with immunotherapy efficacy biomarkers such as immune checkpoints (CD274, CTLA4, LAG3, and PDCD1), T-cell cytotoxicity factors (CD8A, GZMA, GZMB, and IFNG), and epithelial–mesenchymal transition (EMT) biomarkers (CDH1, CDH2, FN1, and VIM) were also calculated. The results indicated that immune checkpoints and T-cell cytotoxicity factors were significantly higher in subtype1, and EMT biomarkers were higher in subtype 2 (**Figure 3B**). The Student’s t-test result suggested that subtype 1 samples were correlated with a higher value of TMB, but the difference was not statistically significant (**Supplementary Figure 2B**).

### The Correlations of Immune Subtypes With Immune Score and Clinical Characteristics

After the calculation of immune scores, stromal scores, and tumor purity scores by ESTIMATE algorithm, S1 samples demonstrated the higher immune and the lower stromal scores, whereas the S2 samples displayed higher stromal and lower immune scores, respectively (*p* < 0.0001, Wilcoxon test, **Supplementary Figures 3A, B**). The ESTIMATE algorithm also revealed that subtype 2 had the higher tumor purity score (*p* < 0.0001, Wilcoxon test, **Supplementary Figure 3C**).

The distribution of datasets and age were not significantly different between the two immune subtypes. Lower-grade tumors (grade 2) were dominant in the S2 TNBC patients, while the higher-grade tumors (grade 3) were predominant in the S1 TNBC patients (**Table 1**). In the tumor–node–mestastasis (TNM) staging system, the rates of the primary tumor (T) (*p* = 0.011) and regional lymph nodes (N) (*p* = 0.029) were significantly different between S1 and S2 samples. Advanced tumors such as T3 and T4 in S2 samples were higher than in S2 samples. However, the differences in rates of distant metastasis (M) and stage between the two subtypes were not significant (**Table 1**).

**Table 1:** Distribution of clinical characteristics among two immune subtypes.

	Subtype 1 (N = 426)	Subtype 2 (N = 508)	*p*-value
**Dataset**			0.534
GSE106977	56 (13.1%)	63 (12.4%)	
GSE18864	20 (4.69%)	18 (3.54%)	
GSE58812	51 (12.0%)	56 (11.0%)	
GSE76124	82 (19.2%)	116 (22.8%)	
GSE76250	72 (16.9%)	93 (18.3%)	
GSE83937	55 (12.9%)	76 (15.0%)	
GSE95700	27 (6.34%)	30 (5.91%)	
TCGA-TNBC	63 (14.8%)	56 (11.0%)	
**Age**			0.273
(26,46)	80 (28.3%)	76 (22.8%)	
(46,55)	76 (26.9%)	84 (25.1%)	
(55,64)	65 (23.0%)	84 (25.1%)	
(64,90]	62 (21.9%)	90 (26.9%)	
**Grade**			<0.001
Grade1	1 (0.66%)	3 (1.60%)	
Grade2	25 (16.6%)	64 (34.0%)	
Grade3	125 (82.8%)	121 (64.4%)	
**Primary tumor (T)**			0.011
T1	27 (19.1%)	37 (21.8%)	
T2	106 (75.2%)	104 (61.2%)	
T3	6 (4.26%)	18 (10.6%)	
T4	2 (1.42%)	11 (6.47%)	
**Regional lymph nodes (N)**			0.029
N0	74 (61.7%)	73 (50.0%)	
N1	35 (29.2%)	40 (27.4%)	
N2	8 (6.67%)	22 (15.1%)	
N3	3 (2.50%)	11 (7.53%)	
**Distant metastasis (M)**			0.624
M0	115 (99.1%)	127 (97.7%)	
M1	1 (0.86%)	3 (2.31%)	
**Stage**			0.320
I	16 (18.6%)	12 (14.3%)	
II	54 (62.8%)	48 (57.1%)	
III	14 (16.3%)	23 (27.4%)	
IV	2 (2.33%)	1 (1.19%)	

### Analysis of DEG and GSEA

**Figure 4:**
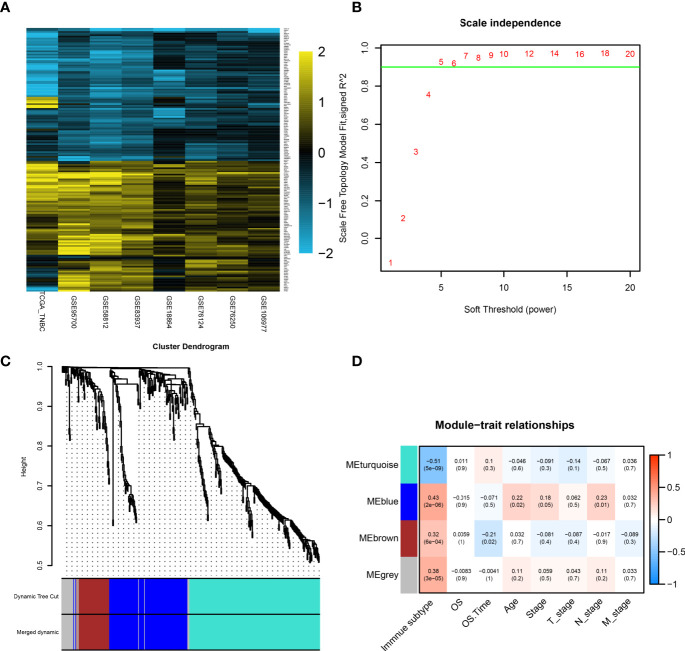
Identification of hub genes by RRA analysis and WGCNA. **(A)** Heatmap showing the top 100 upregulated genes or downregulated genes according to log2 fold change value. Each row represents one gene and each column indicates one dataset. Gold indicates upregulated genes and blue represents downregulated genes in subtype2. **(B)** Analysis of the scale-free fit index for various soft-thresholding powers (β). In all, 5 was the fittest power value. **(C)** The cluster dendrogram of TCGA-TNBC patients. Each branch in the figure represents one gene, and every color below represents one coexpression module. **(D)** PCC matrix between gene module and clinical characteristics. The PCC values range from −1 to 1 depending on the strength of the relationship. A positive value indicates that the genes within a particular coexpression module increase as the clinical trait increases. DEG, differentially expressed gene; RRA, robust rank aggregation; WGCNA, weighted gene coexpression network analysis; PCC, Pearson correlation coefficient.

DEGs between two subtypes were investigated in each cohort (*p* <  0.05 and logFC >  0.5; **Supplementary Figure 4**). The number of upregulated expressed genes in S2 samples were 437 (GSE18864), 1,415 (GSE58812), 467 (GSE76124), 212 (GSE76250), 845 (GSE83937), 760 (GSE95700), 140 (GSE106977), and 2,636 (TCGA-TNBC). The number of upregulated expressed genes in S1 samples were 489 (GSE18864), 1,204 (GSE58812), 686 (GSE76124), 301 (GSE76250), 1,214 (GSE95700), 327 (GSE106977), and 1,223 (TCGA-TNBC). Since no genes could be regarded as DEG in all eight datasets, a total of 440 robust DEGs were determined by the RRA method, including 148 upregulated and 292 downregulated genes in S2. The selected robust DEGs were visualized by the heatmap (**Figure 4A**).

To obtain the enriched pathways related to 440 robust DEGs, GSEA was performed, and the pathways with the lowest *p* value are displayed in **Supplementary Tables 1**, **2**. Some immune-related terms and pathways were largely identified in S1, including immune system process, regulation of immune system process, T-cell receptor signaling pathway, primary immunodeficiency, and adaptive immune system. On the other hand, only one KEGG and two REACTOME pathways were identified in S2, and most of them were related to metabolisms, such as small molecule metabolic process, oxidation regulation process, and lipid metabolic process.

### WGCNA Analysis and PPI Analysis

WGCNA was performed on the expression matrix of 440 robust DEGs of the TCGA-TNBC cohort. According to the scale independence plot, “5” was selected as the best number for soft-thresholding power (**Figure 4B**). Robust DEGs were assigned into different modules based on the degree of coexpression between DEGs. The module containing coexpression genes was given a random color, and the remaining genes were assigned into a gray color module (**Figure 4C**). Correlation between modules and clinical features was calculated and visualized in **Figure 4D**, showing that the turquoise color module possessed a significantly negative correlation with immune subtype (r = −0.51, *p* < 0.01). Since S1 (immune^+^ subtype) and S2 (immune^-^ subtype) as “1” and “2,” the negative correlation with S2 meant the positive correlation with S1. The 196 genes in the turquoise module were selected for further analysis.

**Figure 5:**
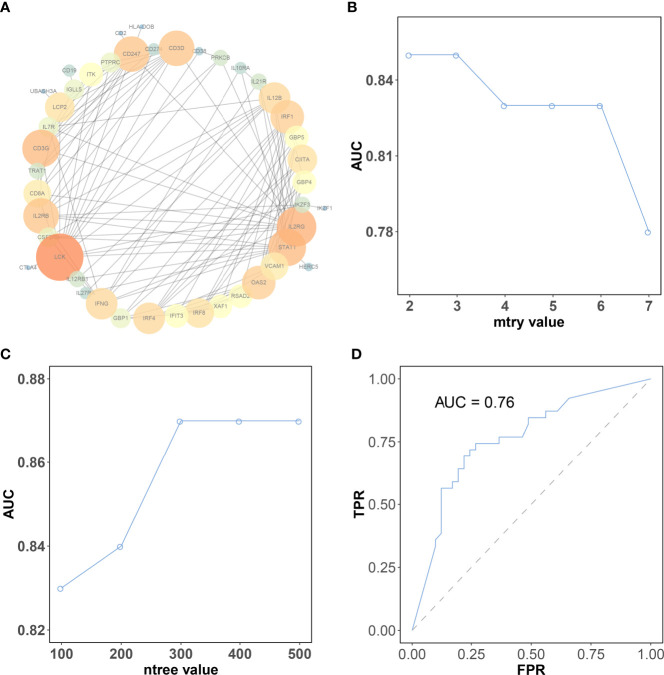
The selection of the best parameter for the machine learning model. **(A)** Protein–protein interaction network of genes in the brown module. The color intensity and the size of nodes were positively correlated with the degree score. **(B)** The “mtry” with the highest AUC was selected as the optimal value of the random forest algorithm. **(C)** The “ntree” with the highest AUC was selected as the optimal value of the random forest algorithm. **(D)** Validation of model in the testing dataset. CR, complete response; PR, partial response; SD, stable disease; PD, progressive disease.

The protein interaction analysis of 196 genes was performed in the STRING database, and the results were visualized by Cytoscape software Based on the criteria of “degree > 10,” a total of 11 hub genes were selected (**Figure 5A**). These 11 hub genes include lymphocyte cell-specific protein-tyrosine kinase (LCK), interleukin 2 receptor subunit gamma (IL2RG), CD3G, signal transducer and activator of transcription 1 (STAT1), CD247, interleukin 2 receptor subunit beta (IL2RB), CD3D, interferon regulatory factor 1 (IRF1), oligoadenylate synthetase 2 (OAS2), interferon regulatory factor 4 (IRF4), and interferon gamma (IFNG). The log2 fold change and *p*-value of 11 hub genes in RRA analysis are provided in **Supplementary Table 3**.

### Construction of Prediction Model of Immune Subtypes

**Figure 6:**
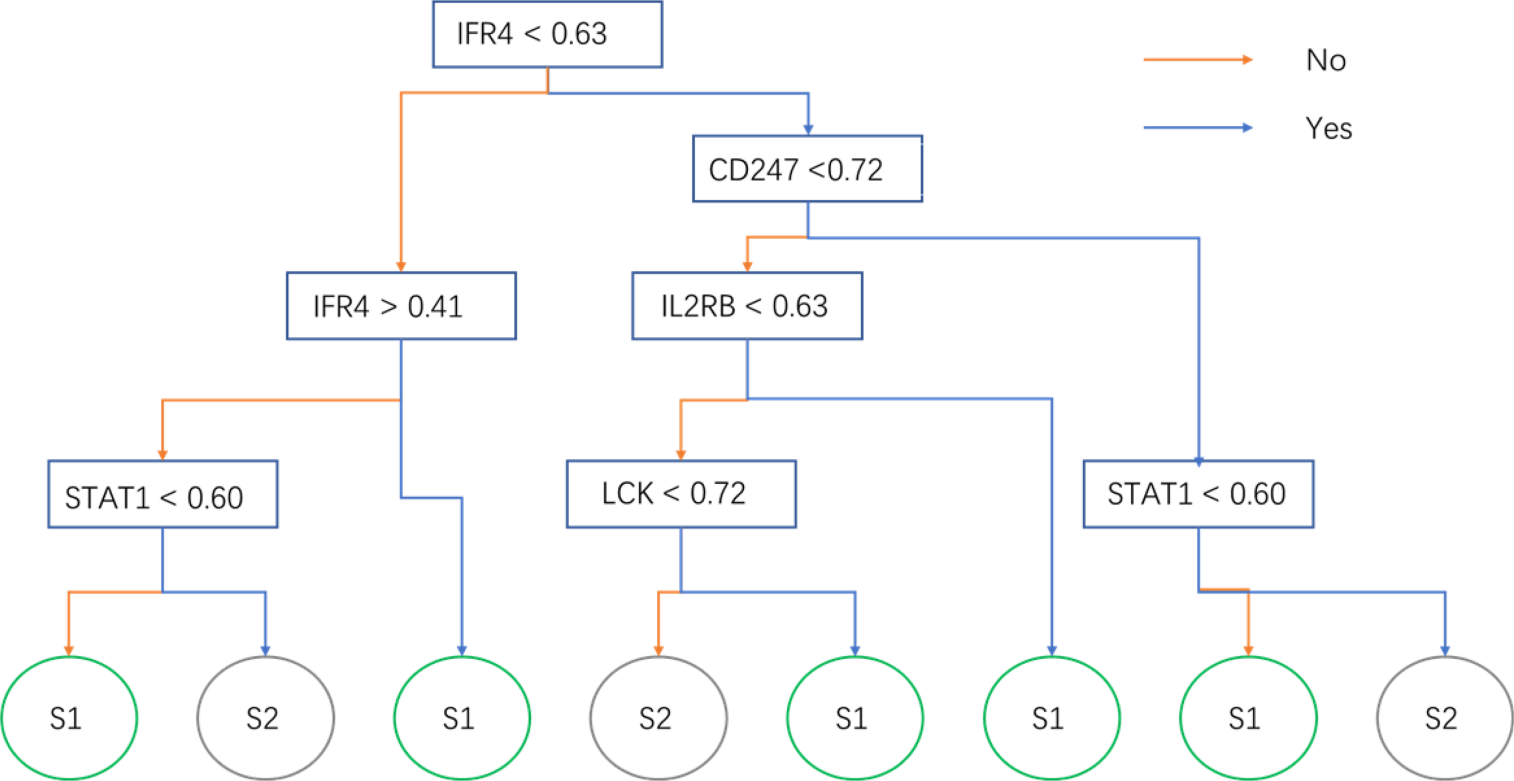
The optimal decision tree in the random forest model. The sample will be predicted into subtypes 1 or 2 by its gene expression.

The expression matrix of hub genes (LCK, IL2RG, CD3G, STAT1, CD247, IL2RB, CD3D, IRF1, OAS2, IRF4, and IFNG) from the TCGA-TNBC dataset was used as input data to build an RF model for immune subtype prediction. The expression values of genes were transformed from numeric values (0–1) into discrete values (“high” or “low”) by the median value. Before RF model construction, the best parameters for the RF model were selected as “mtry = 2” and “ntree = 300” by the best areas under the curve of the receiver operating characteristic (AUC) value (**Figures 5B, C**). After RF model construction, the RF model performed well in the testing dataset indicated with an AUC value of 0.76 (**Figure 5D**). After the construction of the model, the importance of variables is ranked and illustrated in **Supplementary Figure 5**. The most accurate decision tree in the constructed random forest model is shown in **Figure 6**.

### Validation of the Performance of Prediction Model in Independent Datasets

**Figure 7:**
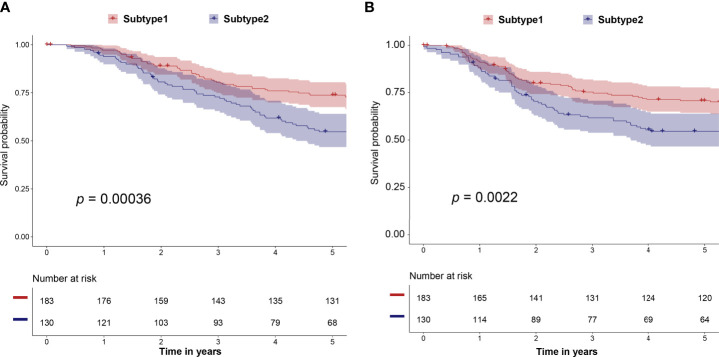
The correlation of predicted immune subtype with the prognosis in the independent dataset (METABRIC dataset). **(A)** Five-year Kaplan–Meier curves for OS of TNBC patients stratified by the immune subtypes. **(B)** Five-year Kaplan–Meier curves for RFS of TNBC patients stratified by the immune subtypes. The *p*-values were calculated by the log-rank test among subtypes. METABRIC, Molecular Taxonomy of Breast Cancer International Consortium; OS, overall survival; RFS, relapse-free survival.

We predicted the immune subtype of these 313 selected samples from the METABRIC dataset. The prediction result demonstrated that the validation dataset contained 183 subtype 1 and 130 subtype 2 samples. The subtype 1 samples in the validation dataset had a better prognosis including OS (*p* = 0.00036) and RFS (*p* = 0.0022) than subtype 2 samples (**Figures 7A, B**). These survival results are consistent with results from the training dataset of TCGA (**Figure 2D** and **Supplementary Figure 2**). Thus, our study identified and validated two robust immune subtypes based on different and independent datasets. Besides, the expression profiles of 10 hub genes were available in the METABRIC dataset. In **Supplementary Figure 6**, high expression of CD3D, CD3G CD247, IL2RG, IRF1, IRF4, LCK, and STAT1 was correlated with better survival. The IFNG and OAS2 showed a similar but not statistically significant tendency.

**Figure 8:**
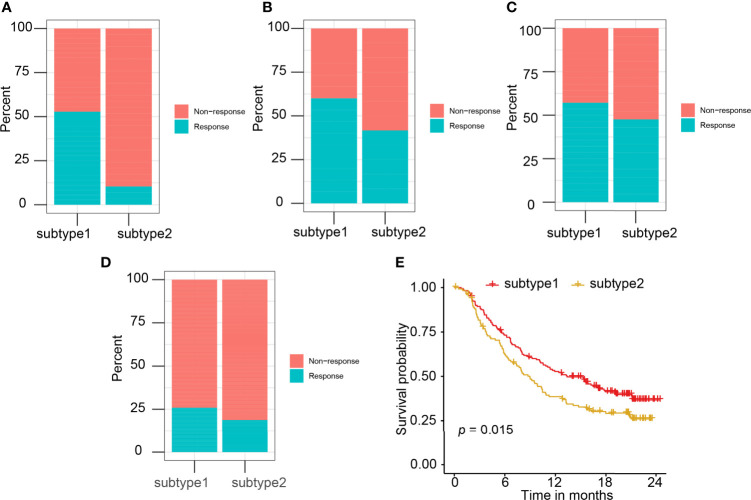
The correlation of predicted immune subtype with the immunotherapy efficacy in the independent datasets. **(A–D)** The correlation of predicted immune subtype with the response rate to immunotherapy in the independent datasets: **(A)** GSE35640, **(B)** GSE78220, **(C)** GSE91061, and **(D)** IMvigor210. **(E)** The correlation of predicted immune subtype with the survival analysis in the IMvigor210 dataset.

Four independent datasets (GSE35640, GSE78220, GSE91061, and IMvigor210), which contain the expression matrix of patients before immunotherapy, were selected as the independent datasets for validating the performance of the immune subtype prediction model. Immune subtypes of patients from these cohorts were predicted by their 11 hub genes (LCK, IL2RG, CD3G, STAT1, CD247, IL2RB, CD3D, IRF1, OAS2, IRF4, and IFNG) expression values. Patients in S1 behaved better overall response rate to immunotherapy than subtype 2 patients (**Figures 8A–D**). The difference in response rates between two immune subtypes in different datasets was 42% in GSE35640 (**Figure 8A**), 20% in GSE78220 (**Figure 8B**), 10% in GSE91061 (**Figure 8C**), and 8% in IMvigor210 (**Figure 8D**). S1 was associated with better overall survival (OS) than S2 (**Figure 8E**). High expression of six hub genes including LCK, CD3G, CD247, IL2RB, IRF1, and IFNG was associated with the better prognosis of patients treated with Atezolizumab (**Supplementary Figure 7**). However, the survival results of CD3G, IL2RG, IRF4, OAS2, and STAT1 were not significant (**Supplementary Figure 8**).

### Web Server Development

**Figure 9:**
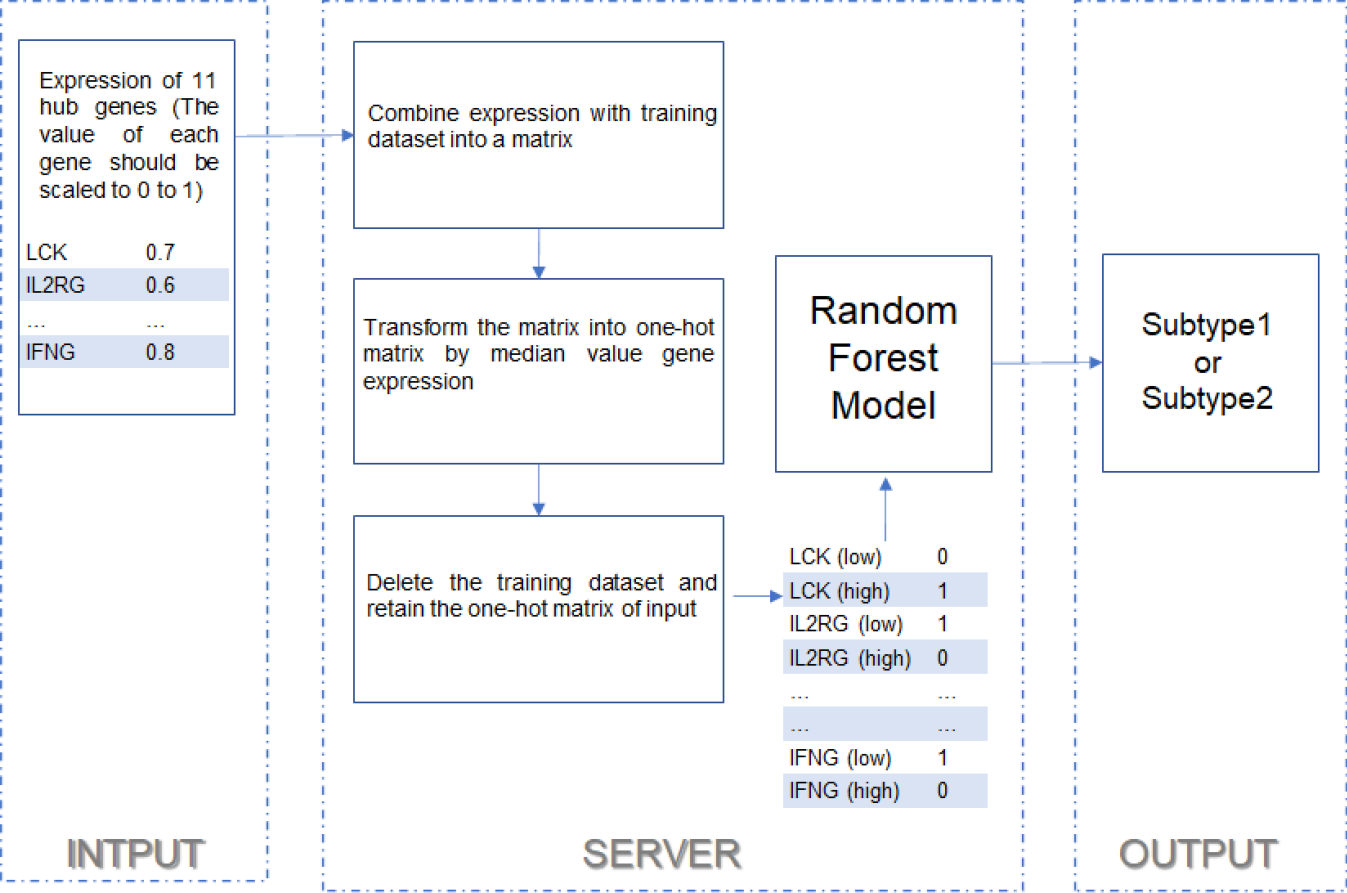
The flowchart of the shiny application.

A web server with the name of triple-negative breast cancer immune subtype (TNBCIS) *via* https://immunotypes.shinyapps.io/TNBCIS/ was built for TNBC immune subtype prediction. The expression data of 11 hub genes (LCK, IL2RG, CD3G, STAT1, CD247, IL2RB, CD3D, IRF1, OAS2, IRF4, and IFNG) will be the input data. Then, the input data will be preprocessed and then used to predict the immune subtype. The flowchart of predicting the TNBC immune subtype is shown in **Figure 9**.

## Discussion

The approval of immunotherapies on the treatment of TNBC brings a new chance to improve the clinical outcomes of TNBC. Multiple clinical trials had demonstrated that the median survival time of TNBC patients in the Immunotherapy–Chemotherapy arm was significantly higher than the Placebo–Chemotherapy arm. However, due to the heterogeneity of TNBC, only approximately 10% of patients are sensitive to immunotherapy. Therefore, clarifying the immune subtype and providing a precise prediction tool have positive significance for screening the dominant populations of immunotherapy. In our research, we carried out the immunophenotyping analysis of a large amount of TNBC samples by unsupervised clustering and built a convenient web tool for clinicians based on machine learning.

The classical biomarkers for a patient decision of treatment immunotherapies include PD-L1 immune cell status, TMB, and tumor-infiltrating T cells, as S1 subtype samples were correlated with a high level of tumor-infiltrating T cells (**Figure 3A**) and TMB (**Supplementary Figure 2B**) immune score (**Supplementary Figure 3A**). This finding prompted us to hypothesize that PD-1/PD-L1 antibodies might be a suitable treatment strategy for S1 subtype patients. The use of these biomarkers is limited because of low prediction accuracy, high costs, and complication. Thus, predicting immune subtype by a small number of genes expression profiles might contribute to the patient decision of treatment immunotherapies. Recently, a 24-gene RNA signature was used to predict immunotherapy effectiveness for gastrointestinal cancer patients (45). Based on six genes, some researchers have constructed a lung cancer risk score model to provide a reference for individualized immunotherapy for patients (46). Some studies construct the prediction model of immunotherapy response for urothelial carcinoma or lung cancer using deep learning of noninvasive radionics biomarkers (47, 48). However, a user-friendly web tool is still not available for TNBC patients. Therefore, the web server constructed in the current study will contribute to the clinical implementation of immunotherapy in TNBC.

Cluster analysis has been widely used in identifying the potential subtypes/subgroups among patients from one dataset. To find the robust immune subtype among TNBC patients, multiple datasets other than a single dataset should be used. However, the batch effect among multiple datasets will be the major barrier to merging different datasets into one dataset. Fortunately, PCA results from **Figures 2A, B** demonstrated that the batch effect was successfully removed after transforming the expression matrix into a matrix of ssGSEA score. Thus, the identified immune subtypes might be more robust than the immune subtype from a single dataset since 931 samples from eight different cohorts were used in our study.

Among the identified immune subtypes, subtype 1 demonstrated higher immune and lower stromal scores and lower tumor purity than subtype 2. These ESTIMATE method results were inconsistent with the result from the ssGSEA method. For example, subtype 1 was enriched in activated CD8 T cells, activated B cells, activated CD4 T cells, and natural killer T cells. Recently, studies advocated tumors could be classified into two categories, namely, “hot” and “cold” (49). Hot tumors demonstrated higher levels of T-cell infiltration and some immune checkpoints such as PD-1 and PD-L1 than cold tumors (49). Hot tumors were correlated with increased response to immunotherapies including PD-1/PD-L1 antibodies (50–52). Patients from S1 would be more likely to respond to immunotherapy, since subtype 1 and S2 identified in this study could be referred to as hot and cold tumors, respectively. Thus, we hypothesized that S1 patients should receive immunotherapy, and S2 patients should not. Consistent with our hypothesis, the results from multiple datasets containing patients treated with immunotherapies also demonstrated that S1 was correlated with a higher response rate and better prognosis to immunotherapy.

The hub genes identified in the current study play crucial roles in the immune system. For example, CD3D, CD3G, and CD247 play an important role in coupling antigen recognition to several intracellular signal-transduction pathways (53). IL2RG and IL2RB are crucial components of T-cell-mediated immune responses (54). OAS2 is a famous innate immune-activated antiviral enzyme for inhibition of viral propagation (55). The interferon regulatory factors (IRFs) including IRF1 and IRF4 are lymphocyte-specific and regulate the activation of innate and adaptive immune systems (56). Moreover, some hub genes have been characterized as biomarkers for immunotherapy efficacy. For example, LCK activity could improve T-cell responses in cancer immunotherapy (57), and STAT1 expression was demonstrated as a potential biomarker for anti-PD-1/anti-PD-L1 for cancer patients (58, 59). The level of IFNG was recognized as a biomarker for lung cancer patients receiving immunotherapies (42).

Before the random forest model construction, the matrix of gene expression was transformed into the matrix of “high” and “low” (**Figure 9**), as the model usually fails to be robust in the validation dataset whose expression ranges from 100 to 10,000 than if the model was constructed in the training dataset whose expression ranges from 1 to 100. It is necessary to ensure that the training and validation dataset have comparable expression value ranges. This transformation was used to increase the robustness of the random forest model. However, the limitations of this study need to be acknowledged. First, the correlation of the proposed immune subtype with the response rate to ICB needs to be validated by TNBC cohorts with ICB treatment. The immune subtype and the constructed predictive model were only tested by melanoma and bladder cancer datasets because the data of TNBC patients with ICB treatment were not available. Second, the mechanisms of these hub genes impacting cancer immunotherapy also need clarification.

## Conclusion

Our study takes full advantage of available TNBC datasets to stratify samples into distinct immunotherapy response subgroups, named S1 and S2. The data from four independent immunotherapy-related cohorts demonstrated that S1 samples had a higher response rate and better prognosis. In addition, a convenient and free web server was provided based on an accurate model constructed in this study. Overall, our study might contribute to explore the heterogeneity among TNBC patients and provide a way to identify the appropriate patient for the immunotherapy.

## Data Availability Statement

The datasets presented in this study can be found in online repositories. The names of the repository/repositories and accession number(s) can be found in the article/**Supplementary Material**.

## Author Contributions

ZC and WS designed and wrote the paper. MW and LT-R collected the related studies and data. ZC, FF, MS, and WS analyzed the data and made the figures and tables. RL and WS revised the manuscript. All authors contributed to the article and approved the submitted version.

## Funding

This study was supported by the University Medicine Oldenburg, Carl von Ossietzky University Oldenburg.

## Conflict of Interest

The authors declare that the research was conducted in the absence of any commercial or financial relationships that could be construed as a potential conflict of interest.

## Publisher’s Note

All claims expressed in this article are solely those of the authors and do not necessarily represent those of their affiliated organizations, or those of the publisher, the editors and the reviewers. Any product that may be evaluated in this article, or claim that may be made by its manufacturer, is not guaranteed or endorsed by the publisher.

## Supplementary Material

Supplementary Figure 1 The selection of best value for the number of immune subtypes. **(A)** Tracking plot for k=2 to 6. In the Tracking plot, the colors in each row represented the samples in different subtypes. **(B)** Consensus clustering cumulative distribution function (CDF) for k=2 to 6. (**c**) Delta area curve of consensus clustering, indicating the relative change in area under CDF curve for each category number k compared with k−1. The horizontal axis represents the category number k, and the vertical axis represents the relative change in area under the CDF curve. CDF, Consensus clustering cumulative distribution function.

Supplementary Figure 2 The correlation of the immune subtype with prognosis and TMB. **(A)** Five-year Kaplan–Meier curves for PFS of TNBC patients stratified by the immune subtypes. *p* value was calculated by the log-rank test among subtypes. **(B)** The distribution of TMB value between two immune subtypes. TMB, tumor mutation burden; PFS, progression-free survival.

Supplementary Figure 3 Differential stromal/immune cell infiltration of the immune subtypes. **(A-C)** Box plots comparing the distribution of stromal/immune cell infiltrations in the two immune subtypes. Each box spans the interquartile range, with the lines representing the median for each group. Whiskers represent the absolute range. All outliers are included in the plot.

Supplementary Figure 4 Volcano plots of the Differentially expressed genes (DEGs) between two subtypes in each dataset. The color of the data points denotes the status of DEGs (red points: highly expressed in subtype2 samples; green points: highly expressed in subtype1 samples).

Supplementary Figure 5 The importance of variables in the random forest model.

Supplementary Figure 6 Ten-year Kaplan–Meier curves for OS of 10 hub genes (from **A–J**: CD3D, CD3G CD247, IFNG, IL2RG, IRF1, IRF4, LCK, OAS2, and STAT1). TNBC patients were stratified by the median value and the expression data came from the METABRIC dataset. *p* value was calculated by the log-rank test among high and low expression groups. METABRIC, Molecular Taxonomy of Breast Cancer International Consortium; OS, overall survival.

Supplementary Figure 7 Five-year Kaplan–Meier curves for OS of 6 hub genes (LCK, CD3G, CD247, IL2RB, IRF1, and IFNG). TNBC patients were stratified by the median value and the expression data came from the IMvigor210 dataset. *p* value was calculated by the log-rank test among high and low expression groups. OS, overall survival.

Supplementary Figure 8 Five-year Kaplan–Meier curves for OS of 5 hub genes (CD3G, IL2RG, IRF4, OAS2, and STAT1). TNBC patients were stratified by the median value and the expression data came from the IMvigor210 dataset. *p* value was calculated by the log-rank test among high and low expression groups. OS, Overall Survival; TNBC, triple-negative breast cancer.

## Abbreviations

ICB, immune checkpoint blockade; TNBC, triple-negative breast cancer; PFS, progression-free survival; CR, complete response; TNBCIS, triple-negative breast cancer immune subtype; BC, breast cancer; FDA, Food and Drug Administration; TMB, tumor mutational burden; TCGA, The Cancer Genome Atlas; DEGs, differentially expressed genes; ssGSEA, single-sample gene set enrichment analysis; ESTIMATE, Estimation of Stromal and Immune cells in Malignant Tumor Tissues Using Expression data; CC, consensus clustering; RRA, robust rank aggregation; GSEA, Gene Set Enrichment Analysis; KEGG, Kyoto Encyclopedia of Genes and Genomes; WGCNA, Weighted Gene Coexpression Network Analysis; GS, gene significance; PPI, protein–protein interaction; RF, random forest; PCA, principal component analysis; OS, overall survival; EMT, epithelial–mesenchymal transition; LCK, lymphocyte cell-specific protein-tyrosine kinase; IL2RG, interleukin 2 receptor subunit gamma; STAT1, signal transducer and activator of transcription 1; IL2RB, interleukin 2 receptor subunit beta; IRF1, interferon regulatory factor 1; OAS2, oligoadenylate synthetase 2; IRF4, interferon regulatory factor 4; IFNG, interferon gamma; AUC, areas under the curve of the receiver operating characteristic (AUC); IRFs, interferon regulatory factors.
