# Supplementary material for: A Machine Learning Model to Predict the Triple Negative Breast Cancer Immune Subtype
Source: Front Immunol. 2021 Sep 17;12:749459. doi: 10.3389/fimmu.2021.749459 (PMC8484710; doi:10.3389/fimmu.2021.749459)
Supplement: Supplementary Figure 1 — The selection of best value for the number of immune subtypes. (A) Tracking plot for k=2 to 6. In the Tracking plot, the colors in each row represented the samples in different subtypes. (B) Consensus clustering cumulative distribution function (CDF) for k=2 to 6. (c) Delta area curve of consensus clustering, indicating the relative change in area under CDF curve for each category number k compared with k−1. The horizontal axis represents the category number k, and the vertical axis represents the relative change in area under the CDF curve. CDF, Consensus clustering cumulative distribution function. [file DataSheet_1.zip › supplement/Supplementary Table1.docx]

| **Pathway** | **Category** | **P.adj** | **NES** | **Gene number** |
| --- | --- | --- | --- | --- |
| IMMUNE SYSTEM PROCESS | GO | 0.034 | -3.45 | 139 |
| REGULATION OF IMMUNE SYSTEM PROCESS | GO | 0.034 | -3.32 | 100 |
| IMMUNE RESPONSE | GO | 0.034 | -3.32 | 100 |
| POSITIVE REGULATION OF RESPONSE TO STIMULUS | GO | 0.034 | -2.16 | 91 |
| DEFENSE RESPONSE | GO | 0.034 | -3.01 | 84 |
| CYTOKINE CYTOKINE RECEPTOR INTERACTION | KEGG | 0.014 | -2.44 | 31 |
| CHEMOKINE SIGNALING PATHWAY | KEGG | 0.014 | -2.4 | 19 |
| T CELL RECEPTOR SIGNALING PATHWAY | KEGG | 0.014 | -2.35 | 12 |
| PRIMARY IMMUNODEFICIENCY | KEGG | 0.014 | -2.52 | 12 |
| CELL ADHESION MOLECULES CAMS | KEGG | 0.034 | -1.73 | 11 |
| REACTOME IMMUNE SYSTEM | REACTOME | 0.015 | -2.69 | 58 |
| ADAPTIVE IMMUNE SYSTEM | REACTOME | 0.015 | -2.23 | 31 |
| SIGNALING BY GPCR | REACTOME | 0.015 | -2.10 | 24 |
| REACTOME GPCR DOWNSTREAM SIGNALING | REACTOME | 0.015 | -2.10 | 24 |
| GPCR LIGAND BINDING | REACTOME | 0.015 | -2.11 | 21 |

**Supplementary Table1.** GSEA results of subtype1. **Abbreviation:** GSEA, Gene set enrichment analysis; NES, normalized enrichment score; P.adj, adjusted P.value.
