# Supplementary material for: A Machine Learning Model to Predict the Triple Negative Breast Cancer Immune Subtype
Source: Front Immunol. 2021 Sep 17;12:749459. doi: 10.3389/fimmu.2021.749459 (PMC8484710; doi:10.3389/fimmu.2021.749459)
Supplement: Supplementary Figure 1 — The selection of best value for the number of immune subtypes. (A) Tracking plot for k=2 to 6. In the Tracking plot, the colors in each row represented the samples in different subtypes. (B) Consensus clustering cumulative distribution function (CDF) for k=2 to 6. (c) Delta area curve of consensus clustering, indicating the relative change in area under CDF curve for each category number k compared with k−1. The horizontal axis represents the category number k, and the vertical axis represents the relative change in area under the CDF curve. CDF, Consensus clustering cumulative distribution function. [file DataSheet_1.zip › supplement/Supplementary Table2.docx]

**Supplementary Table 2.** GSEA results of subtype2. **Abbreviation:** GSEA, Gene set enrichment analysis; NES, normalized enrichment score; P.adj, adjusted P.value.

| **Pathway** | **Category** | **P.adj** | **NES** | **Gene number** |
| --- | --- | --- | --- | --- |
| TISSUE DEVELOPMENT | GO | 0.039 | 2.48 | 38 |
| SMALL MOLECULE METABOLIC PROCESS | GO | 0.039 | 2.72 | 34 |
| OXIDATION REDUCTION PROCESS | GO | 0.039 | 3.17 | 24 |
| LIPID METABOLIC PROCESS | GO | 0.039 | 2.95 | 24 |
| SINGLE ORGANISM CATABOLIC PROCESS | GO | 0.039 | 2.23 | 23 |
| DRUG METABOLISM CYTOCHROME P450 | KEGG | 0.021 | 2.01 | 6 |
| BIOLOGICAL OXIDATIONS | REACTOME | 0.036 | 2.12 | 7 |
| PHASE II CONJUGATION | REACTOME | 0.036 | 2.08 | 5 |
