# Supplementary material for: A Machine Learning Model to Predict the Triple Negative Breast Cancer Immune Subtype
Source: Front Immunol. 2021 Sep 17;12:749459. doi: 10.3389/fimmu.2021.749459 (PMC8484710; doi:10.3389/fimmu.2021.749459)
Supplement: Supplementary Figure 1 — The selection of best value for the number of immune subtypes. (A) Tracking plot for k=2 to 6. In the Tracking plot, the colors in each row represented the samples in different subtypes. (B) Consensus clustering cumulative distribution function (CDF) for k=2 to 6. (c) Delta area curve of consensus clustering, indicating the relative change in area under CDF curve for each category number k compared with k−1. The horizontal axis represents the category number k, and the vertical axis represents the relative change in area under the CDF curve. CDF, Consensus clustering cumulative distribution function. [file DataSheet_1.zip › supplement/Supplementary Table3.docx]

**Supplementary Table 3.** The Log2Foldchange and p.value of hub genes in Robust Rank Aggregation (RRA) Analysis

| **Abbreviation** | **Full name** | **Log2Fold change** | ***p* value** |
| --- | --- | --- | --- |
| LCK | Lymphocyte Cell-Specific Protein-Tyrosine Kinase | -0.81 | <0.001 |
| IL2RG | Interleukin 2 Receptor Subunit Gamma | -0.80 | <0.001 |
| CD3G | - | -0.92 | <0.001 |
| STAT1 | Signal transducer and activator of transcription 1 | -0.83 | <0.001 |
| CD247 | - | -0.61 | <0.001 |
| IL2RB | Interleukin 2 Receptor Subunit Beta | -0.72 | <0.001 |
| CD3D |  | -1.01 | <0.001 |
| IRF1 | Interferon Regulatory Factor 1 | -0.73 | <0.001 |
| OAS2 | Oligoadenylate Synthetase 2 | -0.51 | <0.001 |
| IRF4 | Interferon Regulatory Factor 4 | -0.57 | <0.001 |
| IFNG | Interferon Gamma | -0.76 | <0.001 |
