# Supplementary figures and images for: A Machine Learning Model to Predict the Triple Negative Breast Cancer Immune Subtype
Source: Front Immunol. 2021 Sep 17;12:749459. doi: 10.3389/fimmu.2021.749459 (PMC8484710; doi:10.3389/fimmu.2021.749459)

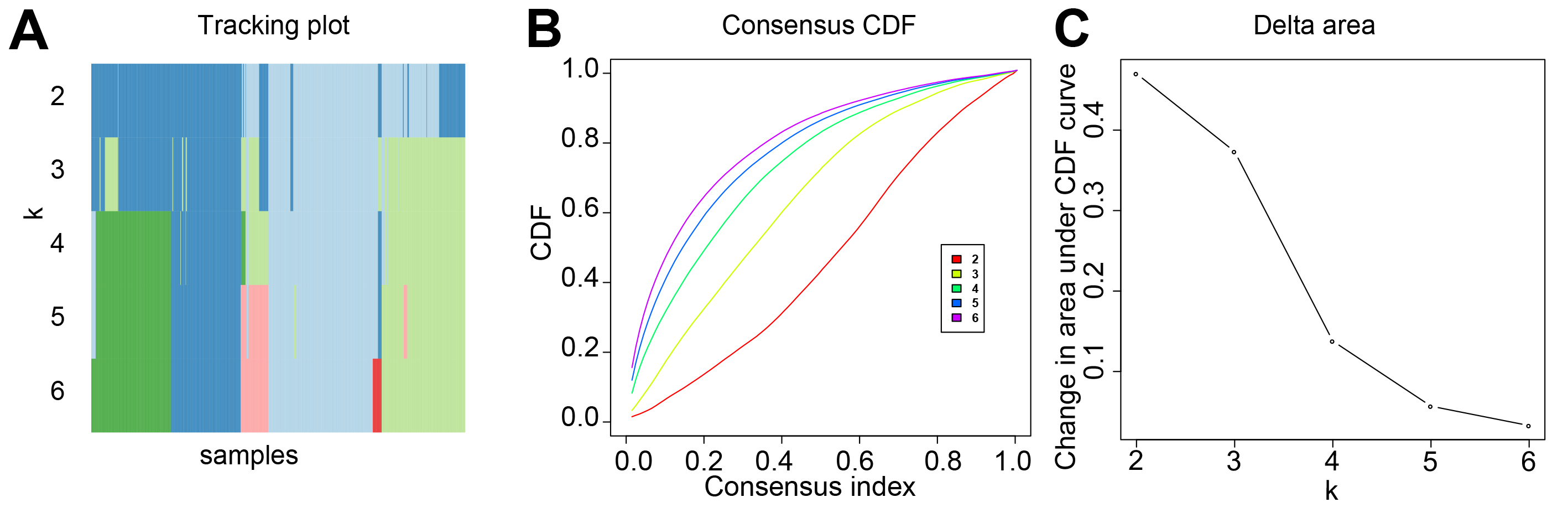

Supplement: Supplementary Figure 1 — The selection of best value for the number of immune subtypes. (A) Tracking plot for k=2 to 6. In the Tracking plot, the colors in each row represented the samples in different subtypes. (B) Consensus clustering cumulative distribution function (CDF) for k=2 to 6. (c) Delta area curve of consensus clustering, indicating the relative change in area under CDF curve for each category number k compared with k−1. The horizontal axis represents the category number k, and the vertical axis represents the relative change in area under the CDF curve. CDF, Consensus clustering cumulative distribution function. [file DataSheet_1.zip › supplement/Supplementary Figure1.tif]

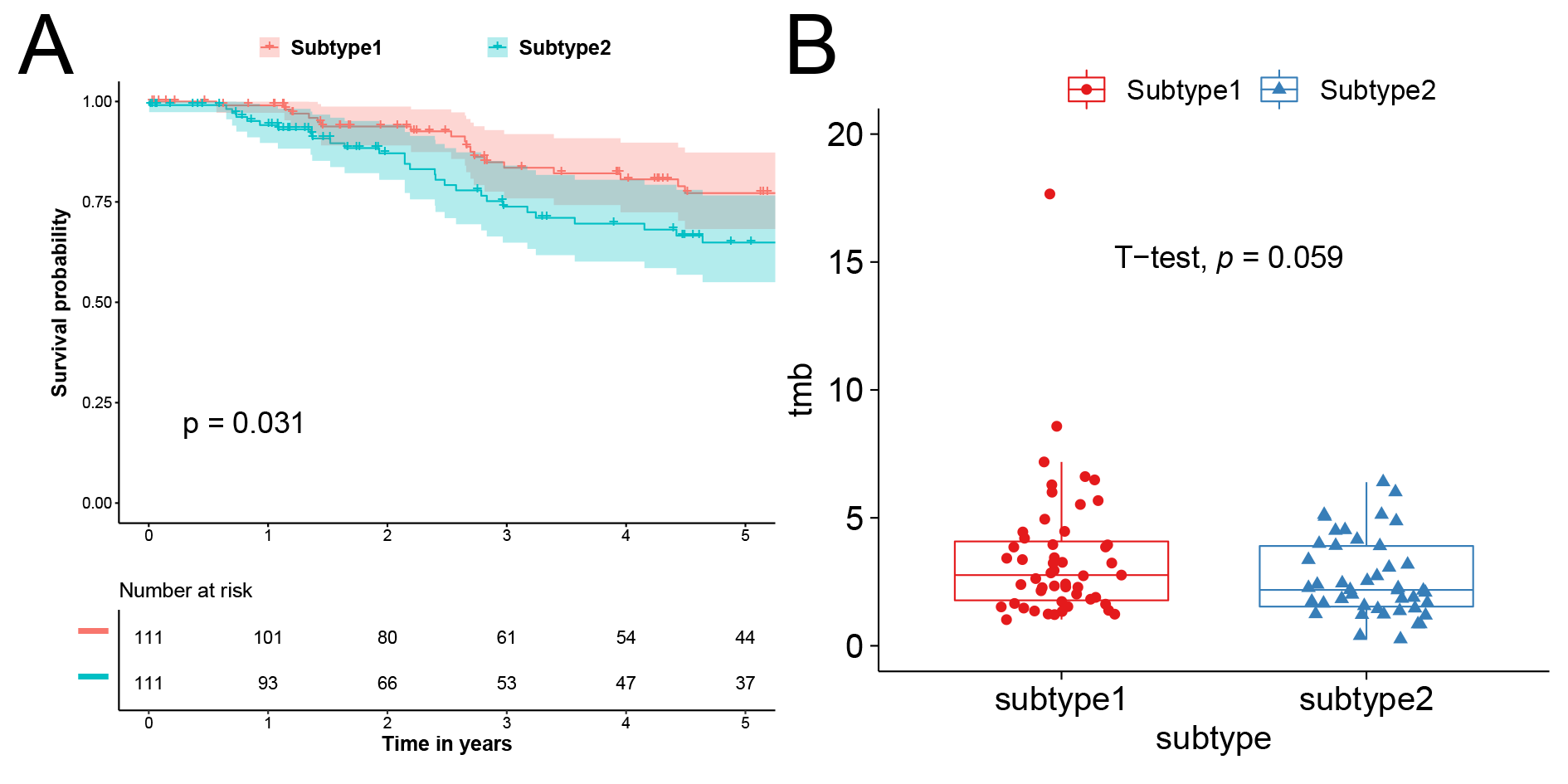

Supplement: Supplementary Figure 1 — The selection of best value for the number of immune subtypes. (A) Tracking plot for k=2 to 6. In the Tracking plot, the colors in each row represented the samples in different subtypes. (B) Consensus clustering cumulative distribution function (CDF) for k=2 to 6. (c) Delta area curve of consensus clustering, indicating the relative change in area under CDF curve for each category number k compared with k−1. The horizontal axis represents the category number k, and the vertical axis represents the relative change in area under the CDF curve. CDF, Consensus clustering cumulative distribution function. [file DataSheet_1.zip › supplement/Supplementary Figure2.tif]

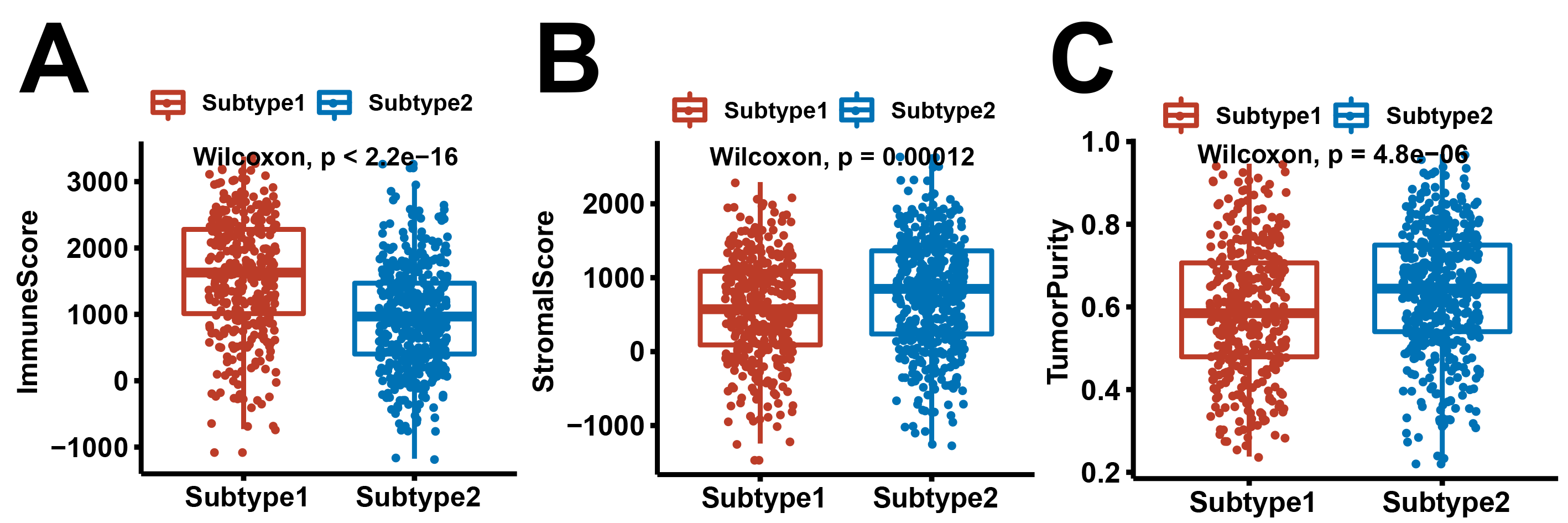

Supplement: Supplementary Figure 1 — The selection of best value for the number of immune subtypes. (A) Tracking plot for k=2 to 6. In the Tracking plot, the colors in each row represented the samples in different subtypes. (B) Consensus clustering cumulative distribution function (CDF) for k=2 to 6. (c) Delta area curve of consensus clustering, indicating the relative change in area under CDF curve for each category number k compared with k−1. The horizontal axis represents the category number k, and the vertical axis represents the relative change in area under the CDF curve. CDF, Consensus clustering cumulative distribution function. [file DataSheet_1.zip › supplement/Supplementary Figure3.tif]

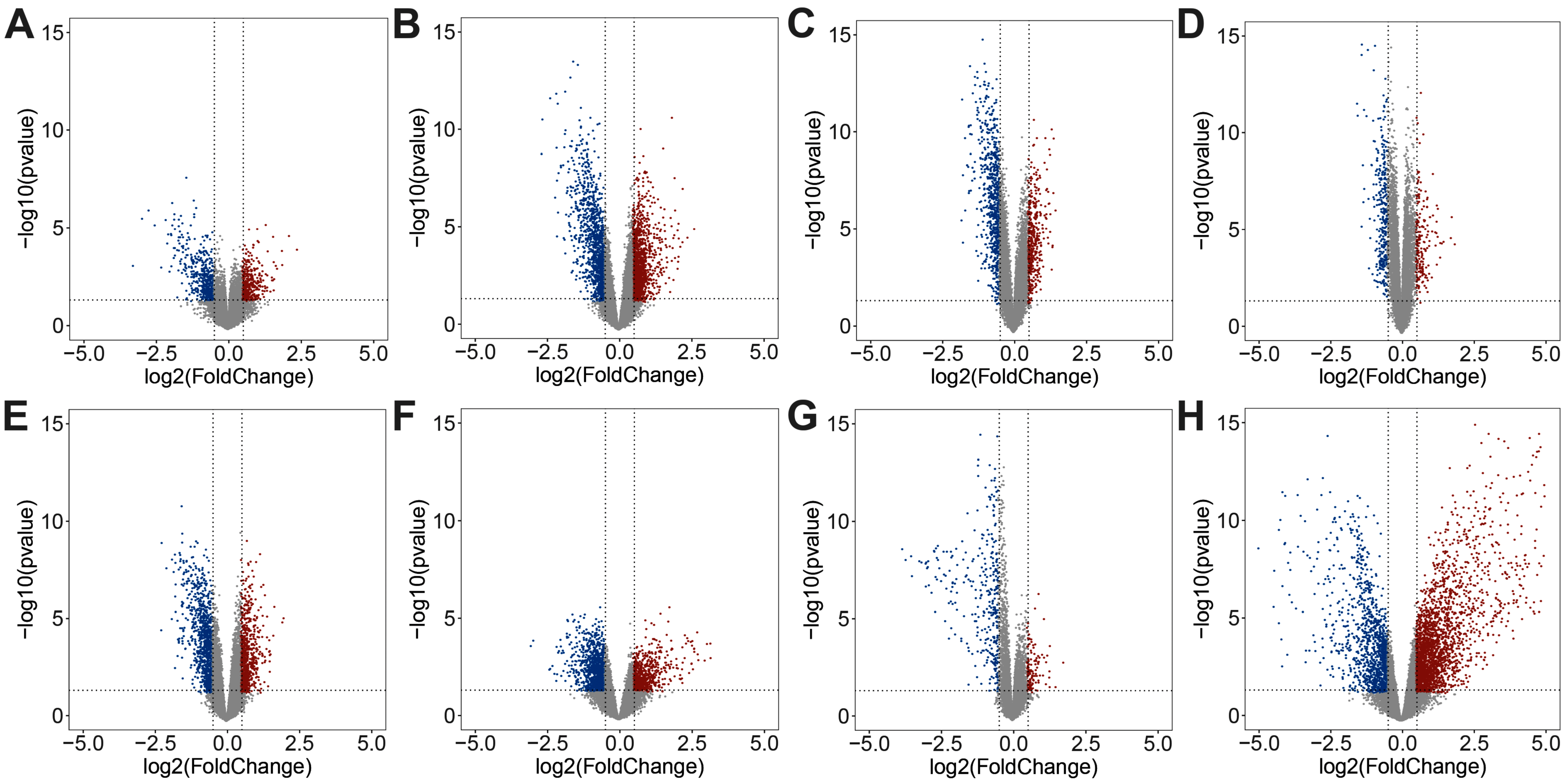

Supplement: Supplementary Figure 1 — The selection of best value for the number of immune subtypes. (A) Tracking plot for k=2 to 6. In the Tracking plot, the colors in each row represented the samples in different subtypes. (B) Consensus clustering cumulative distribution function (CDF) for k=2 to 6. (c) Delta area curve of consensus clustering, indicating the relative change in area under CDF curve for each category number k compared with k−1. The horizontal axis represents the category number k, and the vertical axis represents the relative change in area under the CDF curve. CDF, Consensus clustering cumulative distribution function. [file DataSheet_1.zip › supplement/Supplementary Figure4.tif]

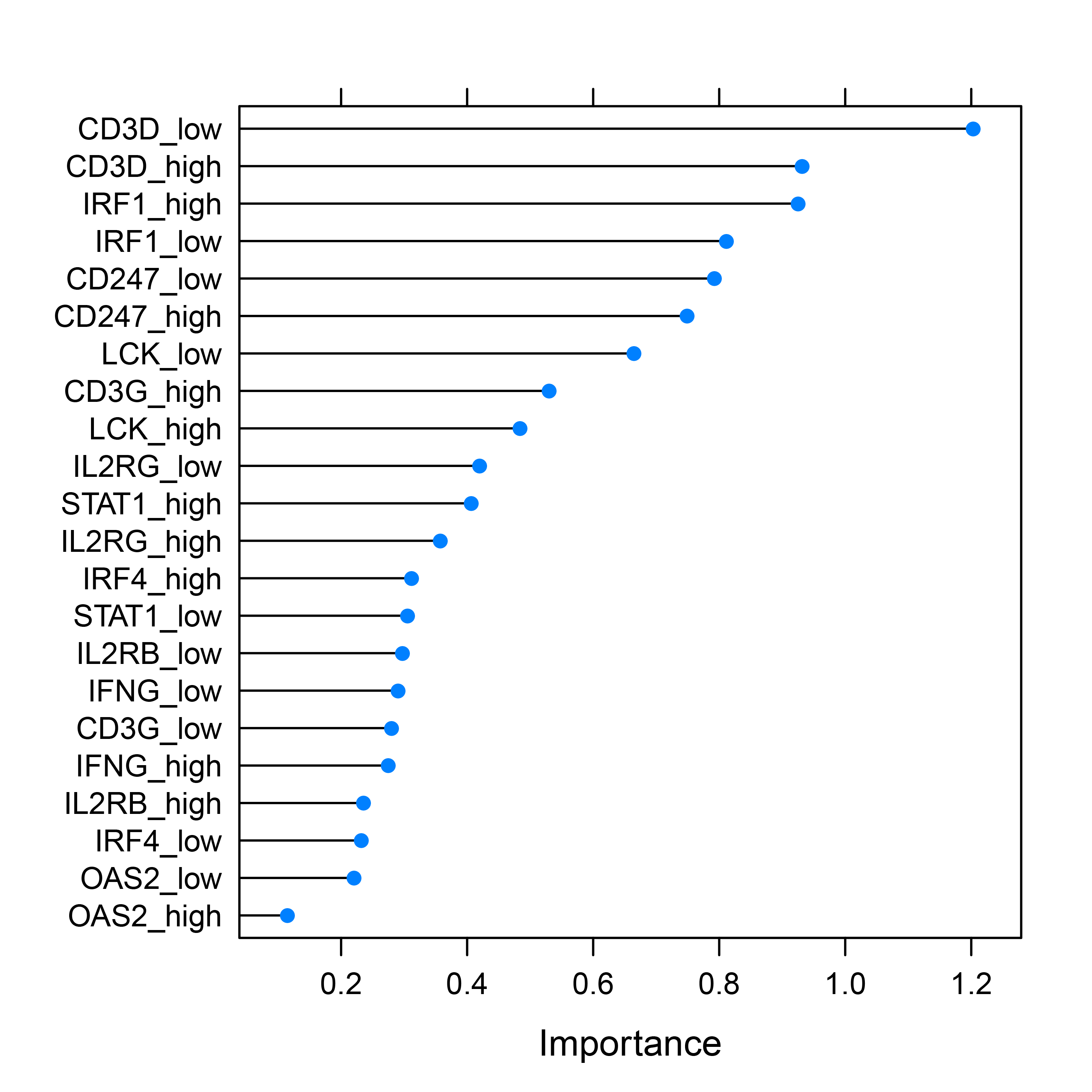

Supplement: Supplementary Figure 1 — The selection of best value for the number of immune subtypes. (A) Tracking plot for k=2 to 6. In the Tracking plot, the colors in each row represented the samples in different subtypes. (B) Consensus clustering cumulative distribution function (CDF) for k=2 to 6. (c) Delta area curve of consensus clustering, indicating the relative change in area under CDF curve for each category number k compared with k−1. The horizontal axis represents the category number k, and the vertical axis represents the relative change in area under the CDF curve. CDF, Consensus clustering cumulative distribution function. [file DataSheet_1.zip › supplement/Supplementary Figure5.tif]

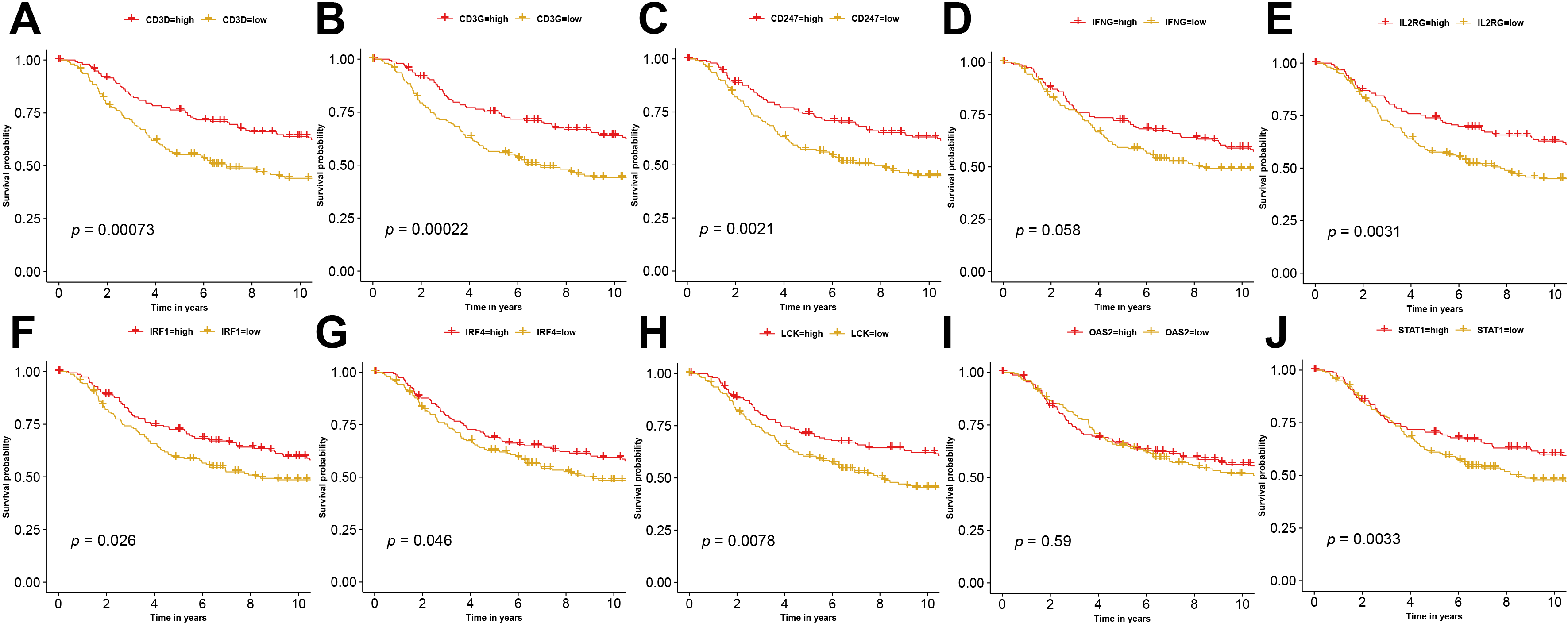

Supplement: Supplementary Figure 1 — The selection of best value for the number of immune subtypes. (A) Tracking plot for k=2 to 6. In the Tracking plot, the colors in each row represented the samples in different subtypes. (B) Consensus clustering cumulative distribution function (CDF) for k=2 to 6. (c) Delta area curve of consensus clustering, indicating the relative change in area under CDF curve for each category number k compared with k−1. The horizontal axis represents the category number k, and the vertical axis represents the relative change in area under the CDF curve. CDF, Consensus clustering cumulative distribution function. [file DataSheet_1.zip › supplement/Supplementary Figure6.tif]

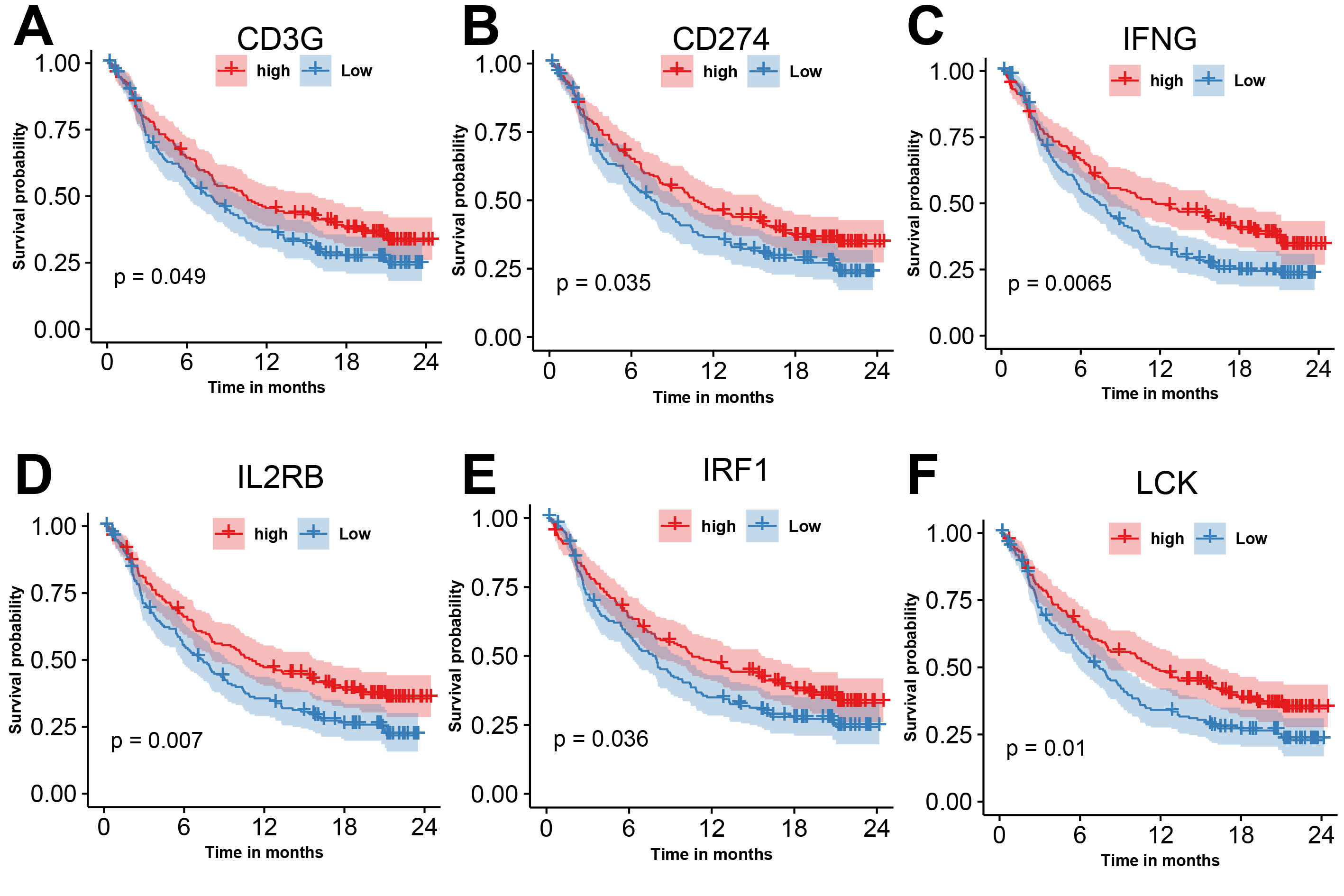

Supplement: Supplementary Figure 1 — The selection of best value for the number of immune subtypes. (A) Tracking plot for k=2 to 6. In the Tracking plot, the colors in each row represented the samples in different subtypes. (B) Consensus clustering cumulative distribution function (CDF) for k=2 to 6. (c) Delta area curve of consensus clustering, indicating the relative change in area under CDF curve for each category number k compared with k−1. The horizontal axis represents the category number k, and the vertical axis represents the relative change in area under the CDF curve. CDF, Consensus clustering cumulative distribution function. [file DataSheet_1.zip › supplement/Supplementary Figure7.tif]

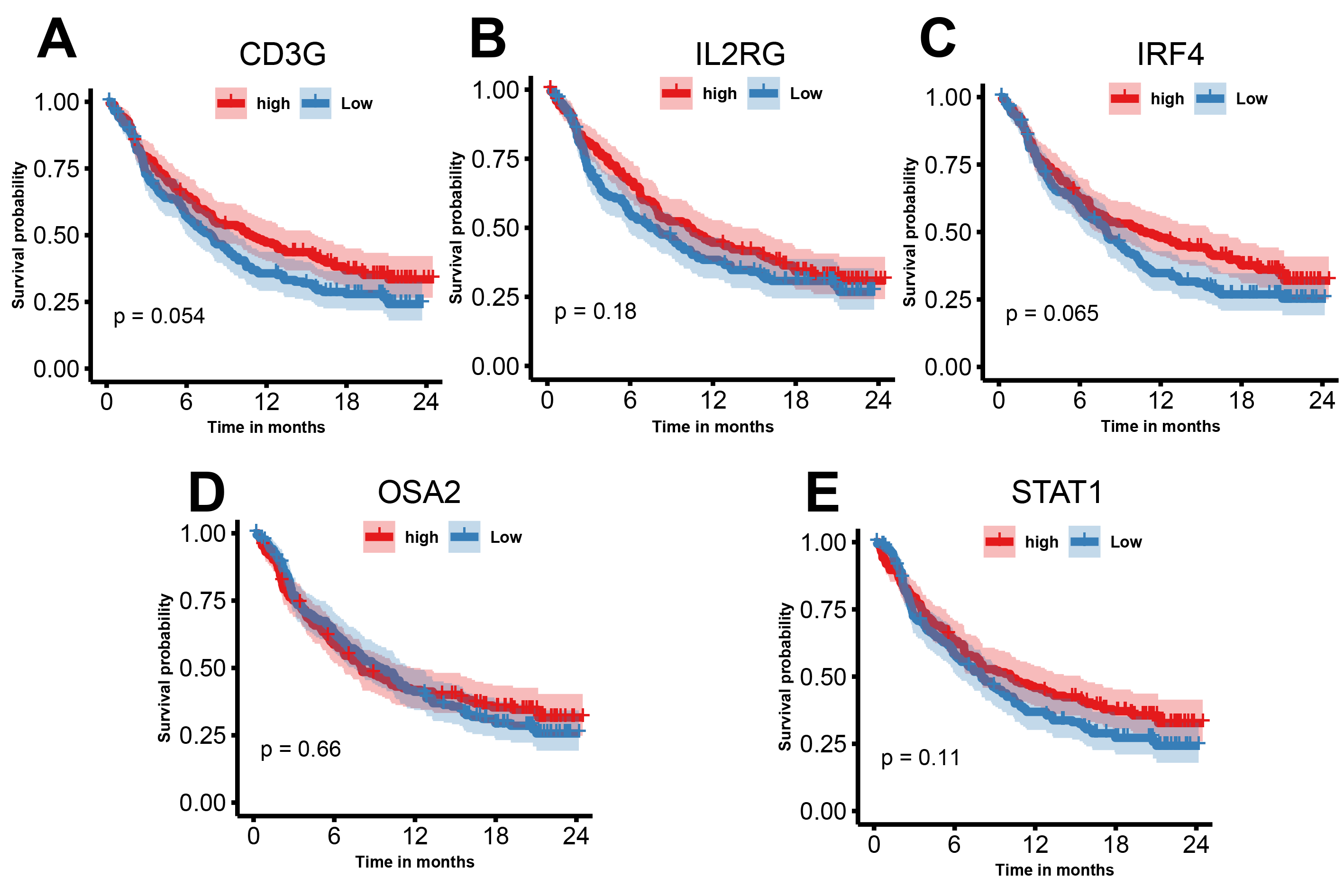

Supplement: Supplementary Figure 1 — The selection of best value for the number of immune subtypes. (A) Tracking plot for k=2 to 6. In the Tracking plot, the colors in each row represented the samples in different subtypes. (B) Consensus clustering cumulative distribution function (CDF) for k=2 to 6. (c) Delta area curve of consensus clustering, indicating the relative change in area under CDF curve for each category number k compared with k−1. The horizontal axis represents the category number k, and the vertical axis represents the relative change in area under the CDF curve. CDF, Consensus clustering cumulative distribution function. [file DataSheet_1.zip › supplement/Supplementary Figure8.tif]
